# Value of P‐wave Parameters in Predicting Outcomes of Repeat Catheter Ablation for Paroxysmal Atrial Fibrillation

**DOI:** 10.1111/pace.15128

**Published:** 2024-12-24

**Authors:** Ibrahim Antoun, Xin Li, Zakkariya Vali, Ivelin Koev, Riyaz Somani, G. André Ng

**Affiliations:** ^1^ Department of Cardiology University Hospitals of Leicester NHS Trust Glenfield Hospital Leicester UK; ^2^ Department of Cardiovascular Sciences Clinical Science Wing University of Leicester Glenfield Hospital Leicester UK; ^3^ Department of research National Institute for Health Research Leicester Research Biomedical Centre Leicester UK

**Keywords:** atrial fibrillation, P‐waves, PTFV1, repeat ablation

## Abstract

**Background:**

Pulmonary vein isolation (PVI) has been established as an effective management option for symptomatic paroxysmal atrial fibrillation (PAF). We aimed to explore the role of P‐wave parameters in a 12‐lead electrocardiogram (ECG) in predicting the success of repeat PAF ablation.

**Methods:**

We enrolled consecutive patients who underwent a second AF ablation procedure for PAF in a UK tertiary center after an index ablation conducted between 2018 and 2019 and a repeat ablation up to 2021. A digital 12‐lead ECG was recorded with a 1–50‐Hz bandpass filter applied. P‐wave duration (PWD), P‐wave voltage (PWV), P‐wave dispersion (PWDisp), and P‐wave terminal force in V1 (PTFV1) were measured before and after the procedure. Changes were correlated with the 12‐month clinical outcome. Procedural success was freedom from ECG‐documented AF up to 12 months following ablation.

**Results:**

Study criteria were satisfied by 72 patients, of which 43 (60%) had successful repeat PVI at 12 months. The mean age is 65, and 47 (65%) were males. The demographics were comparable between both study arms. PWD decreased after successful repeat ablations (136.7 to 124.6 ms, *p* = 0.01) and failed repeat ablations (135.4 to 125.3 ms, *p* = 0.009) without a significant change between both arms. PMV and PWDisp did not change significantly after both study arms. PTFV1 significantly decreased after successful repeat ablations (‐3.1 to ‐4.4 mm.s, *p* = 0.005) without a significant change after failed ablations (‐2.9 to ‐2.7 mm.s, *p* = 0.42). Changes were statistically significant between both arms (*p* = 0.004).

**Conclusion:**

PTFV1 reduction following the second AF ablation was correlated with successful repeat AF ablation at 12 months.

## 1 Introduction

Pulmonary vein isolation (PVI) emerged as the standard of care in symptomatic AF during the last two decades when rhythm control was preferred [1]. The 12‐lead ECG, a traditional clinical tool, may be essential to detecting atrial cardiomyopathy. The normal P‐wave, generated by the atria, has various measured parameters, including duration, morphology, voltage, spatial axis, and area. These parameters can be combined to form a P‐wave index (PWI), such as the morphology‐voltage‐P‐wave duration ECG (MVP ECG) risk score. Changes in these parameters, especially in duration and morphology, can indicate atrial chamber enlargement and conduction blocks and are considered risk factors for clinical events such as AF and ischemic stroke. The predictive value of P‐wave parameters has been recognized for decades, with an advanced interatrial block (IAB) being described in the 1980s as a marker for the risk of AF or atrial flutter [2, 3, 4].

Furthermore, P‐wave parameters, including P‐wave duration (PWD) [5], P‐wave dispersion (PWDisp) [6], P‐wave voltage (PWV) [7], P‐wave terminal force in V1 (PTFV1) [8, 9], and P‐wave area (PWA) [10] have been associated with AF, dementia, stroke and death. Novel P‐wave markers were also correlated with AF ablation failure, including beat‐to‐beat variation, duration‐to‐amplitude ratio, and a notched P‐wave [11, 12, 13]. Modifying the electrical substrate has been suggested to change P‐wave parameters significantly. Hence, they have been used to predict PVI and cardioversion outcomes [14, 15]. These predictions were based on P‐wave parameter correlation with LA remodeling affecting SR maintenance following ablation. The study aims to utilize the P‐wave in the ECG as a non‐invasive measure to predict the 12‐month outcomes of repeat PVI for PAF.

## 2 Material and Methods

This is a single‐center retrospectively observational study involving patients who underwent a second PVI for PAF after a failed first ablation at Glenfield Hospital, Leicester, UK. PAF was defined as AF that terminates spontaneously within 7 days of onset per the European Society of Cardiology guidelines [7]. Inclusion criteria were as follows: patients ≥18 years old who underwent their index procedure between January 2018 and December 2019 requiring a repeat ablation up to December 2021 and completed their 12 months outpatient follow‐up after repeat ablation. Index and repeat procedures were done by radiofrequency without additional ablations outside PVs. Patients on amiodarone were excluded because of its effects on the P‐wave morphology [8]. Patients with pacemakers and those who did not attend a follow‐up were excluded. Furthermore, patients with additional ablations outside the pulmonary veins (PVs) were excluded. Demographics were obtained electronically by examining outpatient clinic letters providing clinical details, medication, ablation details, and follow‐up appointments. Procedure success was defined by the lack of ECG‐documented AF or atrial flutter between 3 months (blanking period) and 12 months following ablation using 12‐lead ECG or ambulatory monitoring. Patients had face‐to‐face clinic follow‐ups in 3, 6‐, and 12 months following ablation with an ECG and symptoms assessment. Patients also underwent 7 days of heart monitoring at the 12‐month mark to ensure the lack of AF recurrence. Furthermore, patients had ECGs should any AF symptoms develop. The study was reviewed and ethically approved by the University of Leicester Research Ethical Committee (REC), reference number 35479‐ia196).

### 2.1 ECG Recordings

Digital 12‐lead ECGs were obtained from every subject before and after repeat ablation. This was archived on the EP LabSystem BARD (Boston Scientific Inc). The system was accessible using computers in the catheter laboratory (cath‐lab). ECGs had a voltage range between 5 and ‐5 mV (range = 10 mV, 16‐bit resolution, band‐pass filtered 1–50 Hz, notch filter). ECGs were exported from LabSystems Pro to an external drive with 60 s of ECG tracing directly before and 60 seconds after ablation in the format of .txt files and imported to MatLab for analysis.

### 2.2 P‐wave Analysis

**FIGURE 1:**
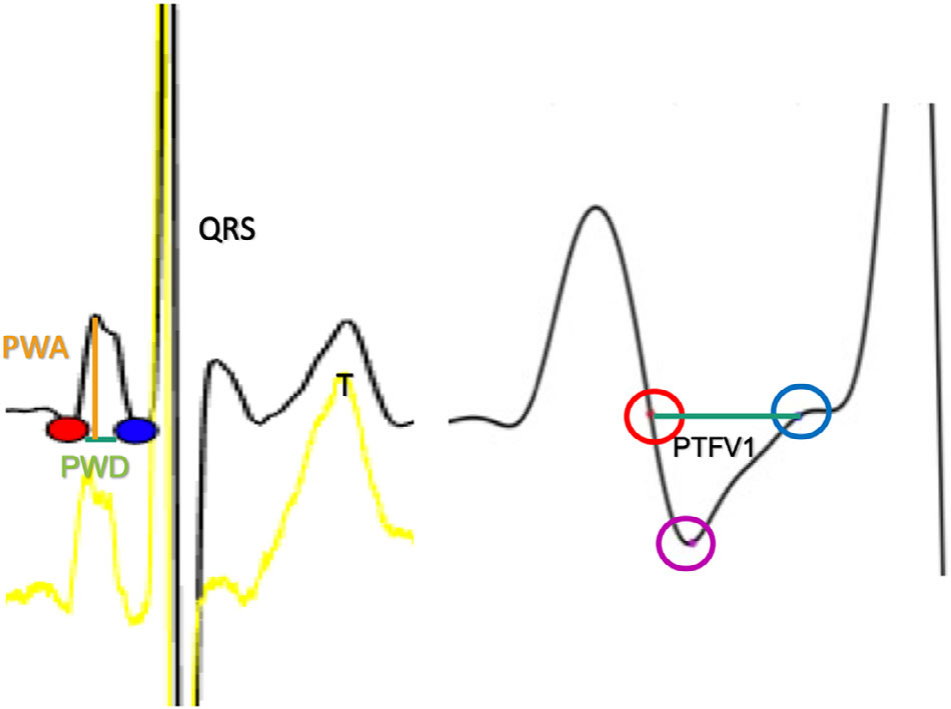
A demonstration of the methodology of electronic P‐wave measurement. The red dot demonstrates the beginning of the P‐wave, while the blue dot demonstrates the end. The purple dot represents the lowest amplitude of the negative phase of the P‐wave in V1. [Colour figure can be viewed at wileyonlinelibrary.com]

The MatLab script allows interactive operations, allowing the user to censor and adjust detected points using the computer. Twenty consecutive P‐wave measurements were averaged to represent a P‐wave pattern in each lead. The P‐wave onset was defined as the point with a minimum perpendicular distance to the line connecting the two T‐Wave and P‐wave peak points. The P‐wave peak was defined as the minimum duration/width of 15 ms in the window of interest. These can be adjusted manually during the measurement process, as demonstrated in Figure 1.

The following P‐wave parameters were produced:

1. PWD: Distance between P‐wave onset and offset. It demonstrates a marker of atrial conduction.
2. PMV: It is measured by the distance from the isoelectric line to the P‐wave peak. It demonstrates atrial voltage.
3. PWDisp: It demonstrates atrial depolarization heterogeneity and is measured by the maximum difference between P‐wave durations.
4. PTFV1: was defined as the product of the maximum absolute amplitude and duration of the negative part of the biphasic P‐wave in (mm.s). It signifies left atrial (LA) depolarization. It was calculated by integrating the P‐wave's negative amplitude with the duration of the negative deflection of the P‐wave.

All 12‐lead parameters were combined in one analysis in each parameter and compared between successful and failed repeat ablation.

### 2.3 Ablation Details

A circular mapping catheter was deployed in the inferior and superior PVs, followed by circumferential ablation of the right‐sided and left‐sided PVs, all guided by three‐dimensional LA mapping (CARTO3, Biosense‐Webster, California, USA). The PVI of the reconnected PVs was expertly conducted using a 3.5‐mm ablation catheter with an externally irrigated tip (ThermoCool SmartTouch Catheter, Biosense‐Webster, California, USA), with ablation index guidance. Post‐procedure PVs dormant conduction was examined using rapid adenosine injection.

### 2.4 Statistical Analysis

Data were stored using Microsoft Excel‐2016, while statistical analysis was conducted using GraphPad Prism V9.3 (San Diego, California, USA). Categorical variables were expressed as frequency and percentage. The mean ± standard error of the mean (SEM) was adopted to describe continuous parametric data. Paired and unpaired *t*‐tests were utilized to analyses matched and unmatched data, depending on the distribution's normality. The normality of distribution was analyzed with the D'Agostino‐Pearson normality test. *p* value ≤ 0.05 represented statistical significance.

### 2.5 Intraobserver Variability Test

There was a human factor in analyzing the P‐wave and manually annotating the P‐wave's start and end. Therefore, intraobserver variability tests were conducted to establish the data's reproducibility. Randomly selected twenty‐two 12‐lead ECGs were analyzed anonymously on 2 consecutive days. 5280 P‐waves were analyzed and compared twice in 2 days. Variability was calculated using raw numbers and a percentage. Results of the intraobserver variability showed the highest variability in PWDisp measurement (4.5 ± 0.3 ms, 19%) followed by PWV (0.03 mV ± 0.001, 13%), PTFV1 (0.4 ± 0.1 mm.s, 10%), and PWD (4.5 ± 0.3 ms, 4%).

## 3 Results

### 3.1 Demographics and Procedure Details

**FIGURE 2:**
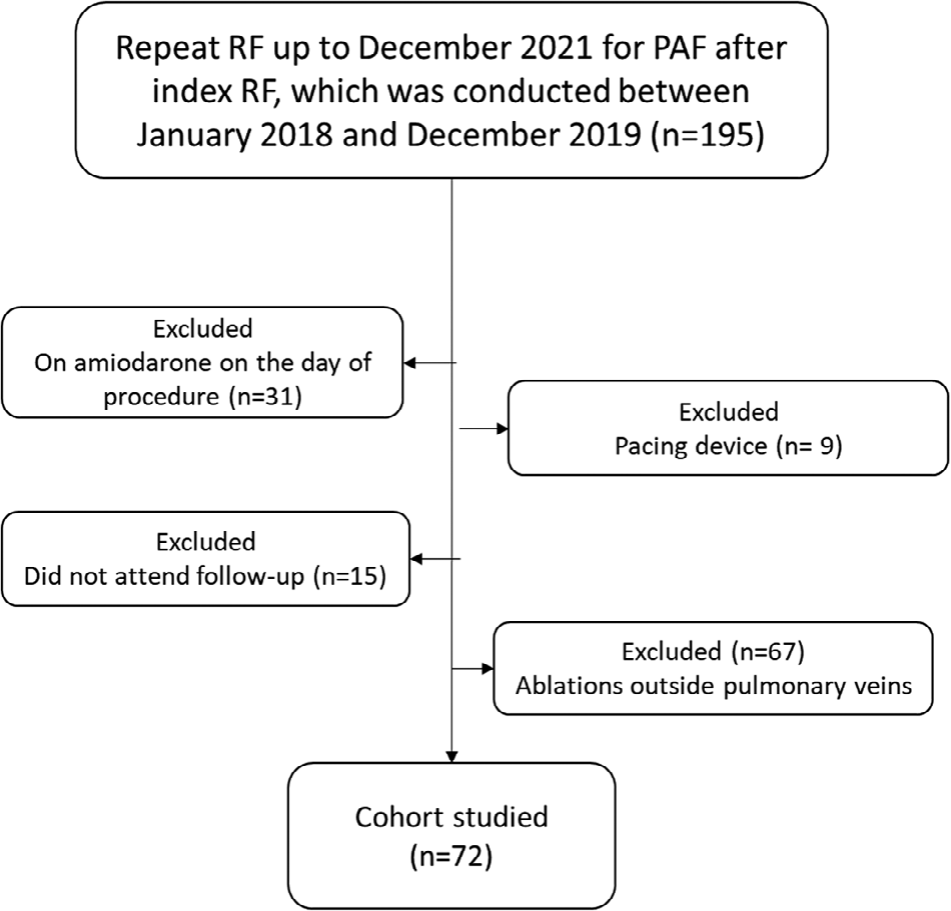
Patient selection flow chart of the study. PVI: pulmonary vein isolation. PAF: paroxysmal atrial fibrillation.

The study criteria were satisfied by 72 patients (Figure 2), of which 43 (60%) had successful repeat PVI at 12 months. Repeat ablations occurred between The mean age was 65, and 47 (65%) were males. Demographics are demonstrated in Table 1. Of the AF recurrences, 7 (24%) were detected on the routine ECG without symptoms, 4 (14%) were detected on the 7‐day monitor at 12 months following ablation, and 18 (68%) had an ECG confirming AF 3 ± 1 days following symptoms. The two groups did not significantly differ in demographics, indexed left atrial volume (LAVI), body mass index (BMI), ablation, and medication details. No patients had low LA voltage in the repeat ablation. All patients had PV reconnections during repeat procedures with a median of two PVs without a statistical difference between both study arms. Furthermore, the proportion of each PV reconnected was similar between both study arms. All PVs were electrically isolated at the end of the procedure, and a bidirectional block was achieved.

**TABLE 1:** Baseline characteristics and ablation details.

	Total (*n* = 72)	Success (*n* = 43)	Fail (*n* = 29)	*p* value
Days between index and repeat procedures	427 ± 54	455.3 ± 40	403.7 ± 68	0.49
Male (%)	47 (65%)	29 (67%)	18 (62%)	0.44
Age (years)	65.2 ± 9	63.5 ± 11	67 ± 11	0.08
Diabetes mellitus (%)	7 (10%)	4 (9%)	3 (10%)	0.99
Heart failure (%)	12 (17%)	7 (16%)	5 (17%)	0.35
Cerebrovascular event (%)	14 (19%)	7 (16%)	7 (24%)	0.54
Ischemic heart disease (%)	15 (21%)	8 (19%)	7 (24%)	0.99
Hypertension (%)	11 (15%)	6 (14%)	5 (17%)	0.45
Left atrium volume indexed (mL/m^2^)	24 ± 2.2	22.1 ± 2.3	26.8 ± 2.2	0.17
Additional ablations (%)	18 (25%)	10 (23%)	8 (28%)	0.76
Body mass index (kg/m^2^)	32.1 ± 1.1	30.2 ± 1.2	33.5 ± 1.5	0.13
Pulmonary veins reconnected and ablated	2.4 ± 1.5	2.3 ± 1.2	2.6 ± 1.4	0.12
Left superior pulmonary vein reconnected	44 (61%)	27 (63%)	17 (59%)	0.64
Left inferior pulmonary vein reconnected	41 (57%)	24 (56%)	17 (59%)	0.75
Right superior pulmonary vein reconnected	46 (64%)	26 (60%)	20 (60%)	0.11
Right inferior pulmonary vein reconnected	35 (49%)	20 (47%)	15 (52%)	0.41
Flecainide after PVI (%)	41 (57%)	23 (53%)	18 (62%)	0.39
Sotalol after PVI (%)	27 (38%)	17 (40%)	10 (34%)	0.11
Time AAD stopped following PVI (months)	5.5 ± 1.3	6.6 ± 1.6	4 ± 0.6	0.38

Abbreviations: AAD, antiarrhythmic drugs; PVI, pulmonary vein isolation.

### 3.2 P‐wave Parameters Changes

**FIGURE 3:**
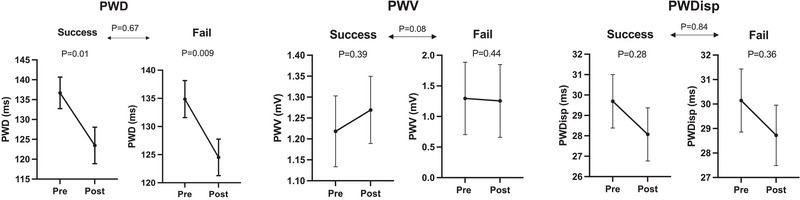
P‐wave duration, P‐wave voltage, P‐wave dispersion before and after repeat ablations. PWD: P‐wave duration. PMV: P‐wave voltage. PWDisp: P‐wave dispersion.

**FIGURE 4:**
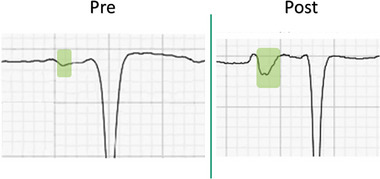
Demonstration of P‐wave terminal force in V1 before and after successful pulmonary vein isolation. [Colour figure can be viewed at wileyonlinelibrary.com]

PWD, PWV, and PWDisp results are demonstrated in Figure 3. PWD decreased after successful repeat ablations (136.7 to 123.5 ms, *p* = 0.01) and failed repeat ablations (135.4 to 125.3 ms, *p* = 0.009). PMV did not change significantly after successful repeat ablation (1.2 to 1.3 mV, *p* = 0.39) and failed repeat ablation (1.3 to 1.3 mV, *p* = 0.44). Similarly, PWDisp did not change significantly after successful repeat ablation (29.7 to 28.2 ms, *p* = 0.28) and failed repeat ablation (30.2 to 28.6 mV, *p* = 0.44). PWD, PWV, and PWDisp changes were insignificant between both arms (*p* = 0.67, *p* = 0.08, *p* = 0.84, respectively). PTFV1 decreased after successful repeat ablations (‐3.1 to ‐4.4 mm.s, p = 0.005) without a significant change after failed repeat ablations (‐2.9 to ‐2.7 mm.s, *p* = 0.42). Changes were statistically significant between both arms (*p* = 0.004). An example of PTFV1 change after successful ablation is demonstrated in Figure 4.

## 4 Discussion

This is the first study to compare PWD, PWV, PWDisp, and PTFV1 directly after repeat ablation with 12‐month outcomes. Our main finding was that a decrease in PTFV1 after repeat AF ablation is associated with successful repeat AF ablation. Patients who experience symptomatic arrhythmia recurrence following initial AF ablation undergo a shared decision‐making process with the clinician regarding the indication for repeat ablation.

PWD decreased regardless of repeat ablation outcome. However, these changes were not predictive of repeat ablation outcomes. The pathogenesis of the reduced PWD, regardless of repeat ablation outcome, remains unclear. One possible hypothesis is that the electrical elimination and conduction of PV sleeves may cause the reduced PWD. Previous studies have demonstrated myocardial sleeves in PVs may be essential in forming the P‐wave  [16, 17]. Ogawa et al. reported that eliminating muscle sleeves inside the PVs resulted in the shortening of the PWD and changes in the terminal portion of P‐wave morphology by analyzing a three‐dimensional computer simulation using an atrial cell model [16]. Another study showed decreased biatrial conduction time after excluding the left PV activations, which significantly correlated with reduced PWD following ablation  [17]. Another hypothesis is that the decrease in PWD seen in both successful and failed ablation likely reflects the immediate procedural impact on atrial conduction rather than long‐term success. Acute inflammation, tissue injury, and temporary atrial remodeling can result in similar changes in P‐wave parameters immediately post‐ablation. Over time, the recurrence of AF in failed ablations may lead to a reversal of these changes. Still, in the short term, both groups can show reduced PWD due to the generalized effects of the ablation process on atrial conduction. However, the PMV may not exhibit a similar change due to the underlying atrial substrate, which can remain unchanged despite the duration reduction. The PMV is influenced by the overall mass of the atrial myocardium, which contributes to the electrical signal and the presence of fibrosis or scarring in the atrial tissue  [18, 19]. Even after successful ablation, areas of low‐voltage due may persist, limiting the potential for PMV  [20]. This suggests that the conduction properties may improve, and the structural changes in the atrial tissue, like fibrosis, can maintain a stable PMV.

Furthermore, atrial late potentials (ALPs) can affect the relationship between PWD and PMV. ALPs are associated with delayed conduction and can contribute to prolonged PWD. Their disappearance post‐ablation can decrease duration without necessarily affecting the PMV, which may remain stable due to structural factors [21, 22].

PTFV1 was first described in 1964 [14] and was correlated with LA volume in 1969 [15]. Earlier studies showed that PTFV1 ←0.04 mm.s was associated with increased AF incidence due to LA remodeling and increased stroke risk due to decreased LA appendage velocity. PTFV1 is altered following AF ablation, reflecting the loss of PV antrum signals, making it relevant before and after AF ablation [16]. PTFV1 is a measure that reflects LA electrical activity, particularly its size and function. A decrease in PTFV1 after successful ablation indicates a reduction in LA strain and potentially a decrease in LA enlargement, often associated with successful restoration of sinus rhythm and improved atrial conduction [22, 23].

The decrease in PTFV1 observed after successful repeat ablation may also reflect the immediate effects of eliminating arrhythmogenic foci and restoring conduction homogeneity rather than extensive reverse remodeling. Although reverse remodeling is generally associated with improved atrial structure and function post‐ablation, it is less likely in this cohort due to the predominance of PAF cases and a preoperative sinus rhythm ECG in many patients. PAF is often associated with less extensive remodeling, where chronic structural and electrical remodeling—such as atrial dilation and fibrosis—is more common than persistent AF.

Therefore, the observed reduction in PTFV1 may result from localized changes in atrial conduction due to PVI rather than a broader structural remodeling effect. This acute improvement in conduction, manifested as decreased LA electrical strain, aligns with findings that PTFV1 reduction can be an early and reliable marker of successful ablation, independent of longer‐term structural changes. However, it is indeed plausible that this change in P‐wave morphology could serve as a surrogate marker for LA structural changes, such as atrial scar formation or conduction disturbances, which may impact the outcome of the ablation [24, 25].

LA scar and conduction abnormalities can lead to electrical heterogeneity within the atrium, influencing P‐wave parameters, including PTFV1. Areas of fibrosis or scarring in the atria can disrupt normal conduction pathways, thereby contributing to prolonged or abnormal P‐wave characteristics that might not directly result from the ablation but instead reflect the underlying atrial substrate. Patients with significant scarring or biatrial conduction disturbances may experience less procedural success due to a less homogenous conduction environment, which is critical for rhythm stability. As such, changes in P‐wave morphology may indirectly correlate with procedural outcomes by reflecting the extent of structural remodeling and fibrosis, which are often markers of atrial remodeling and a greater likelihood of arrhythmia recurrence.

Further studies using advanced imaging techniques, such as cardiac magnetic resonance, to assess LA fibrosis directly would provide additional insights into how P‐wave morphology changes correlate with atrial structural abnormalities. Such investigations could help confirm whether P‐wave morphology changes are indeed primarily reflective of LA scarring and conduction disturbances or if they directly reflect the procedural impact of ablation on atrial conduction [25].

### 4.1 Limitations

This study was not done without its limitations. This is a single‐center retrospective study with AF recurrence detected using 12‐lead ECG or ambulatory monitoring. Long‐term monitoring (implantable loop recorder) was not done, and the AF burden was not evaluated. This could have missed subclinical and micro‐AF episodes. Cardiac magnetic resonance was not utilized to assess LA fibrosis. The relatively low sample size was not derived from formal power calculation. Electroanatomical mapping data of the LA was not obtained. Therefore, a correlation between low voltage areas and PWV was not conducted. Flecainide and sotalol used in our cohort affected PWD. ECGs from the coronary sinus catheter could not be obtained retrospectively and are advised to be obtain in future studies.

Furthermore, patients stopping their antiarrhythmic drugs were included in the analysis. To limit confounding, future studies must match patients with antiarrhythmic drugs and their cessation. One of this study's main limitations is the data's age, and more recent data with the latest ablation methodologies and longer follow‐ups are advised.

## 5 Conclusion

Decreased PTFV1 directly after repeat ablation for PAF is a promising marker for predicting the success of repeat PVI for PAF. More studies are required to confirm these findings.

## Author Contributions

Ibrahim Antoun designed the study, collected the data, conducted the analysis, and wrote the first draft of the manuscript. Xin Li designed the MatLab code to analyze P‐waves parameters. Zakkariya Vali, Riyaz Somani, and G. André Ng reviewed the manuscript before submission. G. André Ng supervised the project.

## Ethics Statement

The study was done on data that was collected routinely and anonymously. It involves human participants. It was approved by the Ethics Review Board of the University of Leicester according to the Helsinki Declaration (reference number: 35479‐ia196), as data was collected retrospectively and anonymously.

## Conflicts of Interest

There is no conflict of interest to declare.

## Data Availability

The authors will make the raw data supporting this article's conclusions available upon reasonable request.
